# Challenging management of a pregnancy complicated by Eisenmenger syndrome; A case report

**DOI:** 10.1016/j.amsu.2021.102721

**Published:** 2021-08-16

**Authors:** Anas Slaibi, Bassel Ibraheem, Farah Mohanna

**Affiliations:** a Department of Pathology, Cancer Research Center at Tishreen University Hospital, Latakia, Syria; b Department of Cardiology at Al-Bassel Hospital, Tartus, Syria

**Keywords:** Case report, Cesarean section, Eisenmenger syndrome, Postpartum management, Pregnancy

## Abstract

**Introduction and importance:**

Women with Eisenmenger syndrome are usually advised to avoid pregnancy because of the high maternal mortality rate of 30–50% which increases up to 65% in the case of a cesarean section. Successful management of Eisenmenger syndrome in pregnancy is tricky and has a narrow margin of safety; however, carefully coordinated multidisciplinary care can profoundly optimize the chances of survival for both mother and baby.

**Case presentation:**

A 28-year-old, 24-week-pregnant patient with a non-corrective ventricular septal defect (VSD) was diagnosed with Eisenmenger syndrome but elected to continue her pregnancy despite the high risks on her and her fetus. Therefore, a multidisciplinary team was assembled to fully monitor the patient and ensure that she reaches 32 weeks before delivery.

**Clinical discussion:**

Multiple scenarios for timing and mode of delivery were discussed. Following the recommendation of the 2018 European Society of Cardiology guidelines and because of the fetus’ transverse position, a cesarean section was performed at week 32 and both the patient and her child were saved.

**Conclusion:**

Termination of pregnancy is the safer option only if it were done early on in the pregnancy. Thus, when the pregnancy is continued, an expert multidisciplinary team is put together to support the patient.

## Highlights

• Women with Eisenmenger syndrome are usually advised to avoid pregnancy.
• The ideal timing and mode of delivery in ES patients with PAH is controversial.
• Older studies suggested that C-section results in higher maternal morbidity and mortality.
• According to recent guidelines, Cesareansections are advised in severe forms of pulmonary hypertension.
• Intensive postoperative monitoring for two weeks is highly recommended.

## 1 Introduction

Eisenmenger's syndrome (ES) was first described in 1897 and was later identified as a congenital heart defect characterized by right to left or bidirectional shunting with severe pulmonary hypertension [1]. ES is uncommon, and it usually occurs in patients over 30 years old [2]. Pregnancy-related physiological changes in Eisenmenger syndrome are poorly tolerated, and they lead to high risk of rapidly progressive cardiopulmonary decompensation, thrombotic complications, and sudden death [3], which is why it is recommended for women with Eisenmenger syndrome to avoid pregnancy or to undergo an early pregnancy interruption within the 10th gestational week [4]. This case report has been reported in line with the SCARE Criteria [7].

### 1.1 Case presentation

A 28-year-old, 24-week-pregnant woman, gravida 1 was referred to the emergency department complaining of a slowly progressive dyspnea over the last month. She had had an uncorrected ventricular septal defect (VSD) at the age of ten, and later, at the age of fifteen, her condition progressed to Eisenmenger syndrome (ES) and was treated with a daily dose (100mg) of Sildenafil. Upon physical examination, she had dyspnea on exertion along with central and peripheral cyanosis. Her oxygen saturation (SpO2) was 60% and blood pressure (BP) 120/70. A transabdominal ultrasound was performed, and the findings were normal (amniotic fluid 700 mL, biparietal diameter (BPD) 59 mm and femur length (FL) 43.5 mm). For further evaluation and monitoring, she was referred to the cardiology department and was placed on continuous oxygen therapy that elevated her SpO2 to 90%, and completely new physical, cardiovascular, and obstetric examinations were performed. Physical examination revealed an SpO2 of 60% at room air BP of 120/70, peripheral and central cyanosis, digital clubbing and no peripheral edema. Auscultation of the chest was clear, and cardiovascular examination showed loud P2 with no murmur and Respiratory rate (RR) of 18 on rest. Echocardiography showed ejection fraction (EF) of 60%, pulmonary artery systolic pressure (PSAP) of 130 mm, a large muscular ventricular septal defect (12mm in diameter) with right-to-left shunting, and dilated right ventricle. The patient elected to continue the pregnancy despite the high risk on her and her fetus. Therefore, a multidisciplinary team consisting of an obstetrician, midwife, cardiologist, anesthetist, intensive care physician and neonatologist was assigned to fully monitor the patient. Our team's original goal was to reach 32 weeks of pregnancy, but because of the patient's low financial status, we decided to admit her at the Cardio department with the following medical plan: bedrest, oxygen administered by mask at 5 L/min, Sildenafil 50mg 1 × 2, Lovenox 40mg 1 × 1, Dopegyt 250mg 1 × 3, and Lasix 40mg 1/2 × 1. Later, we noticed that the patient tolerated her pregnancy well following this regimen; furthermore, her SpO2 levels, EF and PSAP improved grandly which led to a reduction of the dyspnea and cyanosis. At week 28, we started a betamethasone course for fetal lung maturation. At week 30, the patient developed arterial hypertension of 170/90. Preeclampsia and HELLP syndrome were among the major concerns; however, lab tests showed the diagnosis of isolated, pregnancy related hypertension. Thus, Hydralazine was administrated, and the patient was admitted to the cardiac ICU for strict monitoring. When the patient became stable, multiple scenarios for timing and mode of delivery were discussed based on the mother's condition and the fetal position. Risks of vaginal delivery were known to be more endurable than those with cesarean section, but the 2018 European Society of Cardiology guidelines recommend proceeding with a cesarean section in such a case; moreover, the fetus' transverse position rendered vaginal delivery extremely difficult, so we went with a cesarean section. Taking into consideration the state of our patient, we went with epidural anesthesia which was given in the beginning of the surgery with fractionated doses of bupivacaine (65 mg), lidocaine (280 mg), and fentanyl (100 mg) achieving a T-5 anesthesia level after 35 minutes. During the surgery, the patient was asymptomatic. The surgical procedure was uneventful and a female infant of 1,850 g, Apgar score of 9 was delivered. The patient's condition remained stable after delivery, and she was restarted on 7,500 U of subcutaneous heparin which was switched to 40 U of enoxaparin daily on postoperative day 2. She was hospitalized for 1 week and was discharged postoperative day 7 on oral sildenafil 100 mg. Given the risk of further decompensation, she was advised to resume regular close follow-ups after hospital discharge and to implement safe contraception methods.

## 2 Discussion

Eisenmenger's Syndrome is a rare complication of congenital heart disease during pregnancy with a high maternal mortality of 30–50% and even up to 65% in those with Cesarean section [4,5]. Within the first few days postpartum, there is a high risk of sudden death whose major causes could be hypovolemia, thromboembolism and preeclampsia [3,4]. The overall neonatal mortality is 13% and is caused mainly by prematurity. Spontaneous abortion and preterm labor are frequent neonatal morbidity causes whereas intrauterine growth restriction presents only in 30% of pregnancies [6].The presence of Eisenmenger syndrome along with the various hemodynamic changes of pregnancy challenges the brittle cardiopulmonary balance and is regarded as the main cause of cardiopulmonary decompensation [4]. Pregnant women with ES may present with low oxygen saturation, dyspnea, fatigue, dizziness and even right heart failure. Physical examination findings vary from cyanosis, clubbing, and jugular venous distention to mild lower extremity edema. Auscultation may reveal an inspiratory crepitation, loud P2 and a systolic murmur at the pulmonary area [4](1).Restricted antepartum management is necessary when the patient refuses to discuss terminating the pregnancy, or if she presents late in pregnancy. This management includes early hospitalization at 20 weeks of gestation, supplemental oxygen, diuretics, vasodilators and possibly empiric anticoagulation [3]. Maternal arterial oxygen tension should be kept at ≥70 mmHg as it decreases the blood flow across the right-to-left shunt and thereby improves oxygen saturation in patients with ES [4]. Moreover, in the third trimester, oxygen is provided by mask at 5 L/min to improve the patient's hypoxic condition and reduce pulmonary artery pressure [1]. When congestive symptoms are present, loop diuretics may be added with caution because maintaining effective cardiac output needs a critical cardiac preload [3](1), and they may lead to hypovolemia and resulting hypotension, which may worsen right ventricular function. Early initiation or continuation of PDE-5 inhibitors such as sildenafil in pregnancy is recommended [3]. On the other hand, for patients at risk of preterm labor, dexamethasone is prescribed to decrease the risk of respiratory distress syndrome and mortality as premature infants born at <32 weeks' gestation are at significant risk of surfactant deficiency. In normal pregnancies, the physiological decrease of protein S along with the increase in fibrinogen and other prothrombotic factors predispose patients to thrombosis. Additionally, ES may enhance this prothrombotic state of pregnancy and lead to micro-embolisms with severe consequences. Nonetheless, the evidence supporting anticoagulant therapy in patients with ES was insufficient, and it has been suggested that anticoagulants would increase risk of hemoptysis [1](3). Consequently, on a case-by-case basis, weight-adjusted prophylactic dosing of low-molecular-weight heparin (LMWH) or unfractionated heparin (UH) can be considered. LMWH is often preferred over UH as it is easier to administrate and has a lower risk of heparin-induced thrombocytopenia (HIT) [3].

The ideal timing and mode of delivery in ES patients with PAH is controversial. Older studies suggested that elective cesarean section carries no maternal benefit and results in earlier delivery while vaginal delivery is associated with less blood loss and lower risk of infection [4,5]. Additionally, cesarean section results in higher maternal morbidity and mortality (75%) than vaginal delivery (34%) [3](1). However, according to the 2018 European Society of Cardiology guidelines for the management of cardiovascular diseases during pregnancy, C-sections are advised in severe forms of pulmonary hypertension (including ES). Advantages of cesarean delivery are that the time and opportunity of delivery can be controllable, and the presence of senior staff can usually be ensured. Because our patient had ES with high PAH, and her fetus was in a transverse position, a C-section was inevitable. A carefully executed anesthetic plan can decrease some of the potential side effects of labor and surgical delivery. There are multiple anesthetic techniques; however, taking into consideration the conditions of our case, epidural anesthesia was chosen as it alleviates perioperative pain and reduces the pulmonary and systemic vascular resistances thus causing less tachycardia, less myocardial oxygen consumption and reduction of the right-to-left shunting [4].Nowadays more pregnant women with ES survive because medicine and neonatal care are developing. The first postpartum week is considered a period of maximum mortality; thus, intensive postoperative monitoring is necessary as most hemodynamic changes of pregnancy resolve by two weeks postpartum [5,6]. Fetal outcome is closely related to the hematocrit level. For a successful pregnancy the hematocrit should be lower than 65% and the arterial oxygen tension higher than 70 mmHg. When arterial oxygen saturation is maintained higher than 90% in pregnancies with ES, 92% of the fetuses survive, but when oxygen saturation fell below 85%, survival falls to 12% [1].

## 3 Conclusion

Termination of pregnancy is the safer option only if it were done early on in the pregnancy. Thus, when the pregnancy is continued, an expert multidisciplinary team is put together to support the patient. Proactive serial clinical assessment, intensive daily monitoring and a closely followed, well-planned medical regiment are the keys to achieve a successful delivery. The mode and time of delivery need to be personalized; caesarean section is indicated for patients with severe forms of pulmonary hypertension according to the 2018 European Society of Cardiology guidelines.

## Guarantor

Farah Mohanna, MD.

## Patient perspective

The patient participated in the treatment decision and he was satisfied with the results of the treatment after identifying the final diagnosis.

## Patient consent form

Written informed consent was obtained from the patient for publication of this case report. A copy of the written consent is available for review by the Editor-in-Chief of this journal on request.

## Sources of funding

There are no sources of funding.

## Ethics approval

No ethical approval necessary.

## Registration of research studies

Not applicable. Our manuscript is a case report.

## Author contributions

Anas Slaibi conceived the study, analyzed and interpreted the patient data and drafted the final manuscript. Bassel Ibrahim contributed in the treatment and follow up of the pagathering patient information and drafting the manuscript.

Farah Mohanna supervised the whole project, reviewed the final manuscript and was a major contributor in writing the manuscript.

## Research registration

N/A.

## Provenance and peer review

Not commissioned, externally peer-reviewed.

## Declaration of competing interest

We have no conflict of interest.

## Registration of research studies

Not applicable. Our manuscript is a case report.

## Annals of medicine and surgery

The following information is required for submission. Please note that failure to respond to these questions/statements will mean your submission will be returned. If you have nothing to declare in any of these categories then this should be stated.
