# Supplementary material for: Challenging management of a pregnancy complicated by Eisenmenger syndrome; A case report
Source: Ann Med Surg (Lond). 2021 Aug 16;69:102721. doi: 10.1016/j.amsu.2021.102721 (PMC8379476; doi:10.1016/j.amsu.2021.102721)
Supplement: Multimedia component 2 [file mmc2.pdf]

CARE
case report guidelines

CARE Checklist of information to include when writing a case report

(cc) BY-NC-ND

✓

Topic	Item	Checklist item description	Reported on Line
Title	1	The diagnosis or intervention of primary focus followed by the words "case report"	. .lines 2,3. . .
Key Words	2	2 to 5 key words that identify diagnoses or interventions in this case report, including "case report" .	line 32 . .
Abstract (no references)	3a	Introduction: What is unique about this case and what does it add to the scientific literature?	.lines 17-22 . . . . .
	3b	Main symptoms and/or important clinical findings	. .lines 22-24 . . . .
	3c	The main diagnoses, therapeutic interventions, and outcomes	. lines 25-28 . . . .
	3d	Conclusion—What is the main "take-away" lesson(s) from this case?	. .line 21. . . . .
Introduction	4	One or two paragraphs summarizing why this case is unique (**may include references**)	lines 36-43
Patient Information	5a	De-identified patient specific information.	lines 46-49 .
	5b	Primary concerns and symptoms of the patient.	. lines 50-52
	5c	Medical, family, and psycho-social history including relevant genetic information	lines 48-50 .
	5d	Relevant past interventions with outcomes	. lines 48,49
Clinical Findings	6	Describe significant physical examination (PE) and important clinical findings.	lines 51-54, 58-62
Timeline	7	Historical and current information from this episode of care organized as a timeline	N/A
Diagnostic Assessment	8a	Diagnostic testing (such as PE, laboratory testing, imaging, surveys).	. lines 51-53, 59- 62,
	8b	Diagnostic challenges (such as access to testing, financial, or cultural)	lines 66, 71, 72
	8c	Diagnosis (including other diagnoses considered)	. lines 48,49, 74
	8d	Prognosis (such as staging in oncology) where applicable	.N/A
Therapeutic Intervention	9a	Types of therapeutic intervention (such as pharmacologic, surgical, preventive, self-care)	lines 80-84
	9b	Administration of therapeutic intervention (such as dosage, strength, duration)	lines 67, 68, 74, 86-88
	9c	Changes in therapeutic intervention (with rationale)	lines 89-90
Follow-up and Outcomes	10a	Clinician and patient-assessed outcomes (if available)	. lines 87-90
	10b	Important follow-up diagnostic and other test results	.N/A
	10c	Intervention adherence and tolerability (How was this assessed?)	.lines 87-90
	10d	Adverse and unanticipated events	lines 72-74
Discussion	11a	A scientific discussion of the strengths AND limitations associated with this case report	lines 91-140
	11b	Discussion of the relevant medical literature **with references**.	.lines 138-142
	11c	The scientific rationale for any conclusions (including assessment of possible causes)	lines 125-133
	11d	The primary "take-away" lessons of this case report (without references) in a one paragraph conclusion . .	.N/A
Patient Perspective	12	The patient should share their perspective in one to two paragraphs on the treatment(s) they received .	.N/A
Informed Consent	13	Did the patient give informed consent? Please provide if requested	Yes ☑ No ☐
